# Supplementary material for: Oscillating light engine realized by photothermal solvent evaporation
Source: Nat Commun. 2022 Sep 24;13:5621. doi: 10.1038/s41467-022-33374-x (PMC9509359; doi:10.1038/s41467-022-33374-x)
Supplement: Supplementary file 1 — Supplementary Information [file 41467_2022_33374_MOESM1_ESM.pdf]

Supplementary Information

Oscillating Light Engine Realized by Photothermal Solvent Evaporation

Jingjing Li[1,2], Linlin Mou[3], Zunfeng Liu[3*], Xiang Zhou[1*],Yongsheng Chen[3*]

[1]Department of Science, China Pharmaceutical University, Nanjing 211198, China
[2]College of Chemistry and Chemical Engineering, Anyang Normal University, Anyang, Henan 455000, China
[3]State Key Laboratory of Medicinal Chemical Biology, Key Laboratory of Functional Polymer Materials, Frontiers Science Center for New Organic Matter, College of Chemistry, Nankai University, Tianjin 300071, China
E-mail:liuzunfeng@nankai.edu.cn, zhouxiang@cpu.edu.cn, yschen99@nankai.edu.cn

This PDF file includes:

Supplementary Note 1. Light-to-work energy conversion efficiency (Page 2)

Supplementary Note 2. Natural frequency of the PP/CB oscillators (Page 2)

Supplementary Figures 1 to 38 (Pages 3–21)

Supplementary Table 1 (Page 22)

Supplementary References (Page 23)

Supplementary Notes

Note S1: Light-to-work energy conversion efficiency

The light-to-work energy conversion efficiency (η) can be given by:

$$\eta = \frac{E_{\text{out}}}{E_{\text{in}}} \tag{1}$$

where E_{in} is the incident light energy, E_{out} is mainly the elastic energy.

The E_{in} and E_{out} were calculated by the following equations:[1]

$$E_{in} = PAt \tag{2}$$

$$E_{\text{out}} = \frac{Ehlw^3}{24\kappa^{-2}} \tag{3}$$

where P is the light intensity (800 mW cm^{-2}), A is the surface area of the PP/CB actuator infiltrated with ethanol (0.38 cm^2), t is the response time (0.167 s). E is the modulus (0.28 MPa). h, l, w, and κ is the thickness (148.5 μm), length (10.62 cm), width (0.36 cm), and curvature (7.3 cm^{-1}), respectively. All the parameters used in the calculations were obtained after the PP/CB film was immersed in ethanol. The incident light energy and elastic energy are calculated to be about 51 mJ and 0.46 mJ, respectively. So that the energy conversion efficiency is about 0.9%.

Note S2: Natural frequency of the PP/CB oscillators

The natural resonant frequency f of the PP/CB film was defined as follows:[2]

$$f = \frac{\alpha^2}{2\pi}\sqrt{\frac{EI}{\rho A l^4}} = \frac{\alpha^2}{2\pi}\sqrt{\frac{\frac{1}{12}Ewh^3}{\rho whl^4}} = \frac{\alpha^2}{4\pi}\sqrt{\frac{Eh^2}{3\rho l^4}} \tag{4}$$

$$I = \frac{1}{12}wh^3, \quad A = wh \tag{5}$$

In the equation (1), α is 1.875, which depends on the oscillation mode. *I, E and A* represent the moment of inertia, modulus and sectional area of the film. Besides, the l, w, and h represent the length, width and height of the film immersed with or without ethanol, respectively. The ρ are calculated to be about 0.13 and 0.67 g cm^{-3}, when the 100 μm film was immersed with or without ethanol, and the calculated oscillation frequency is about 3.9 and 6.3 Hz. When the film generates oscillation under NIR irradiation, the obtained experimental frequency is between 3.3 and 6 Hz, having a good agreement with the theoretical data.

Supplementary figures

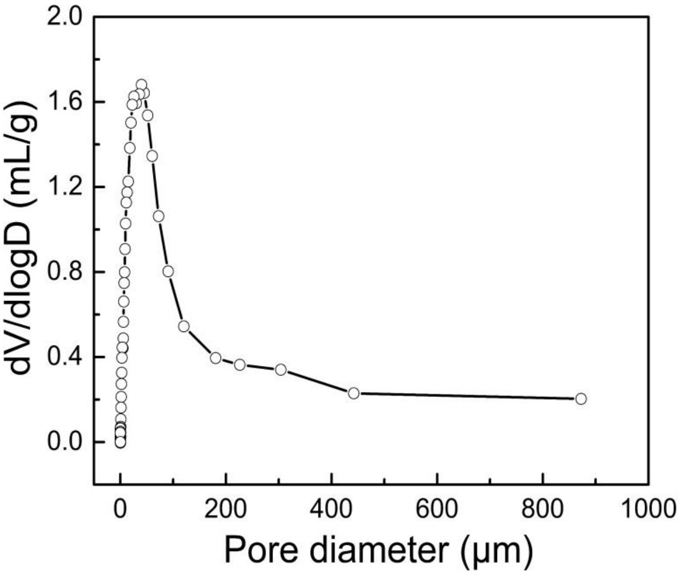

Supplementary Fig. 1. Pore size distribution of the porous PP/CB film measured by the mercury intrusion method.

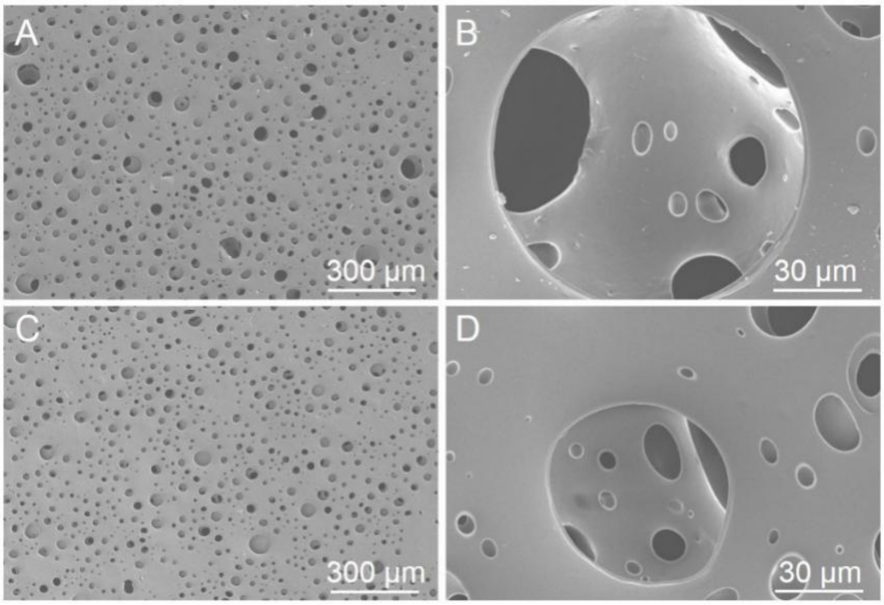

Supplementary Fig. 2. Low and high magnifications for the porous PP/CB film with thickness of 60 μm (A, B) and 150 μm (C, D).

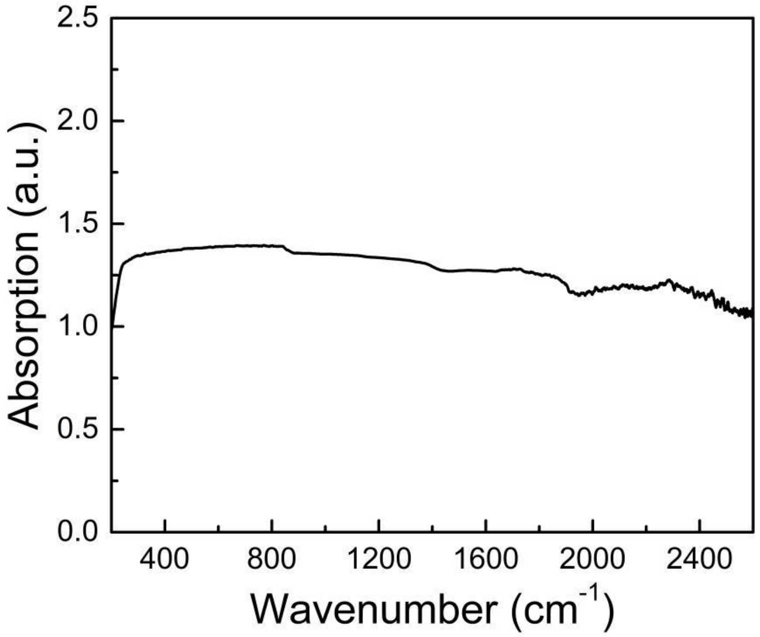

Supplementary Fig. 3. Absorption spectra of the PP/CB film.

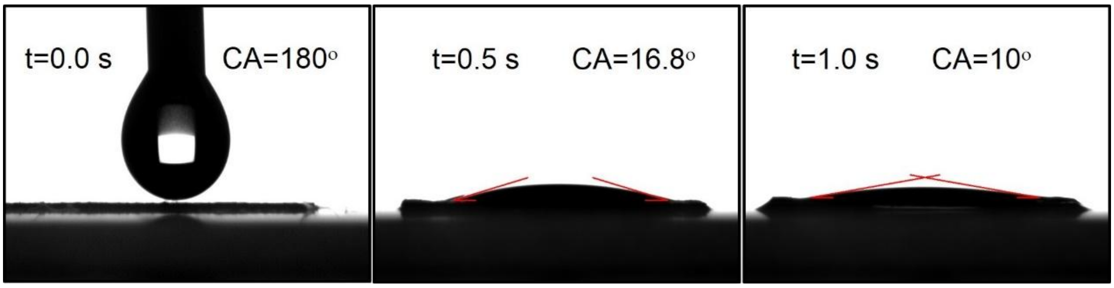

Supplementary Fig. 4. Photographs at different time during contact angle measurement by dropping ethanol on the porous PP/CB film with thickness of 100 μm.

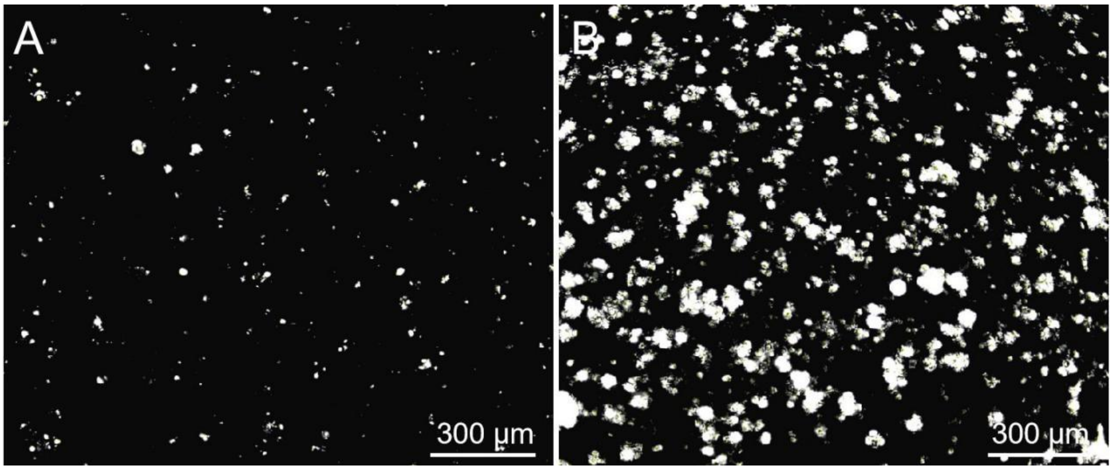

Supplementary Fig. 5. Optical images of the porous PP/CB film before (A) and after (B) infiltrated with ethanol.

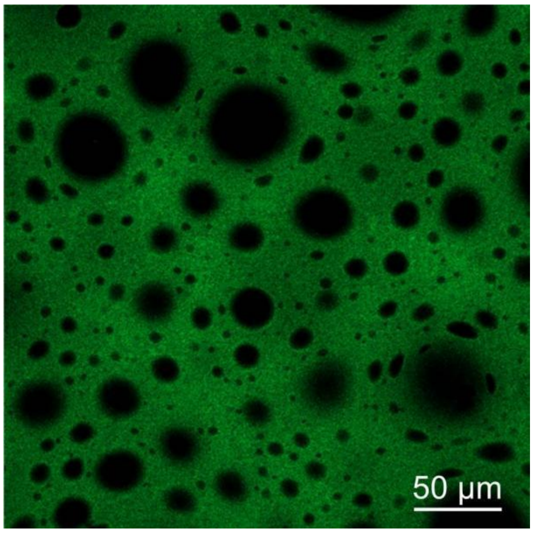

Supplementary Fig. 6. Fluorescent image of the 100 μm thickness porous PP/CB film infiltrated with rhodamine/ethanol solution (0.5 mg mL^{-1}).

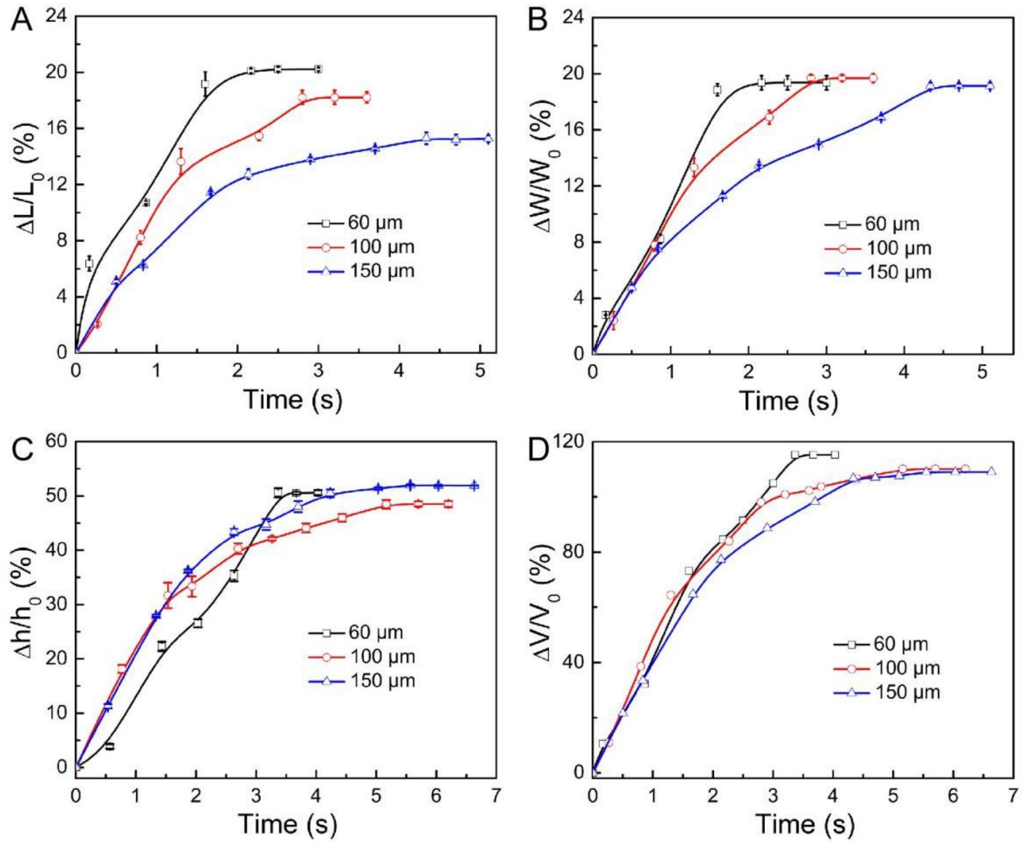

Supplementary Fig. 7. Change ratio in length (A), width (B), thickness (C), and volume (D) as a function of time for the porous PP/CB film with different thickness by absorbing ethanol. The original size of the PP film is 3 cm × 1 cm × 100 μm. Error bars denote the standard deviation.

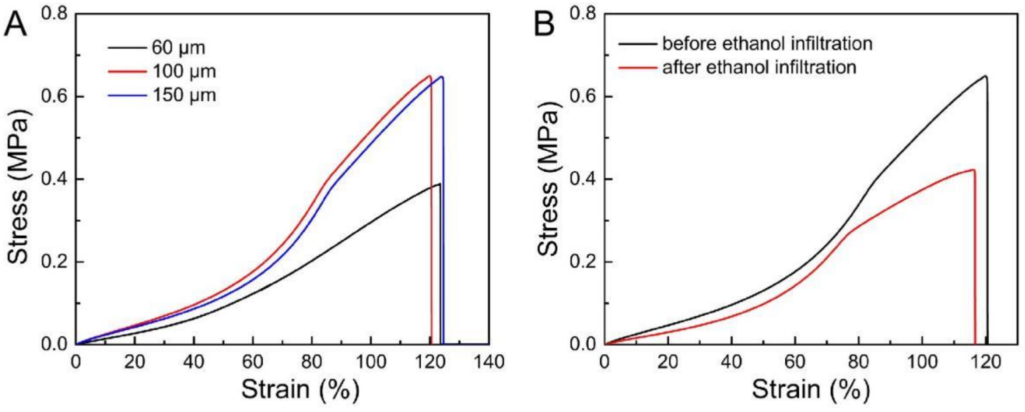

Supplementary Fig. 8. Stress-strain curves for the porous PP/CB film (A) with different thickness, and (B) before and after infiltration of ethanol. The stretch rate is 0.5% s^{-1}. The original size of the PP film is 2 cm × 1 cm × 100 μm.

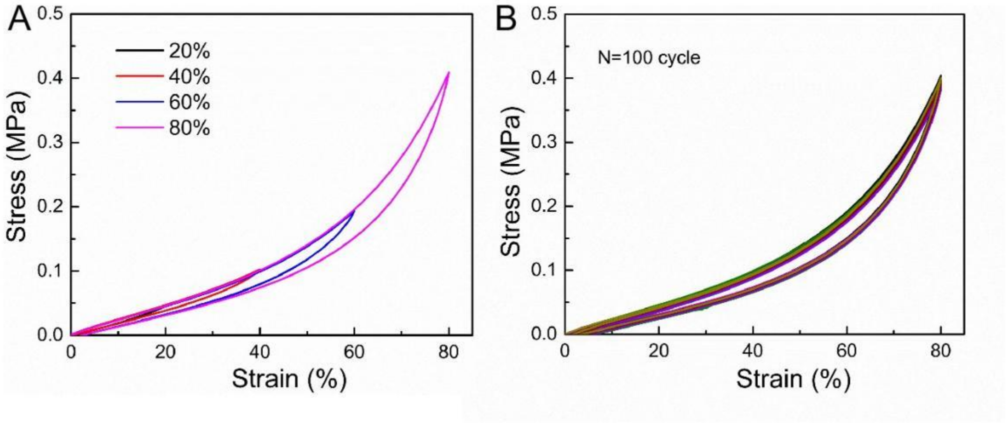

Supplementary Fig. 9. Stress-strain curves with progressively increasing strain (A) and cyclic stress-strain tests (B) for the porous PP/CB film. The stretch rate is 0.5 % s^{-1}. The original size of the PP/CB film is 2 cm × 1 cm ×100 μm.

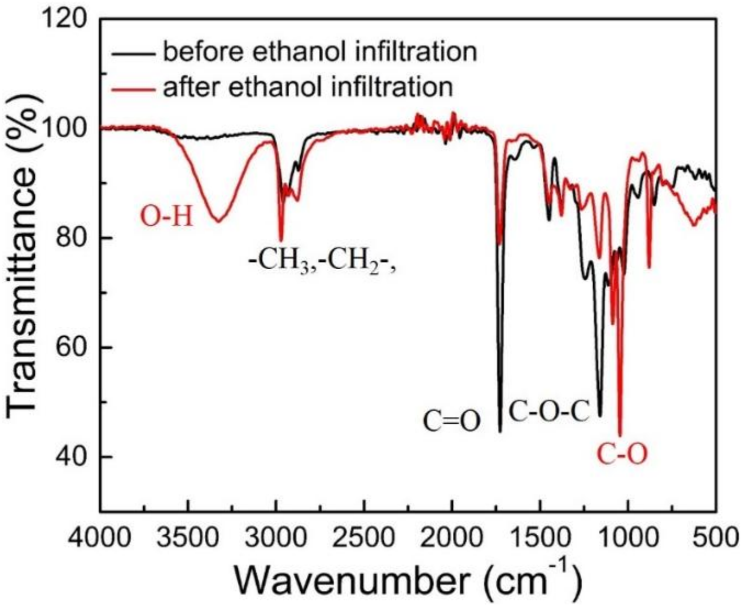

Supplementary Fig. 10. FTIR spectra for the porous PP/CB film before and after ethanol filtration.

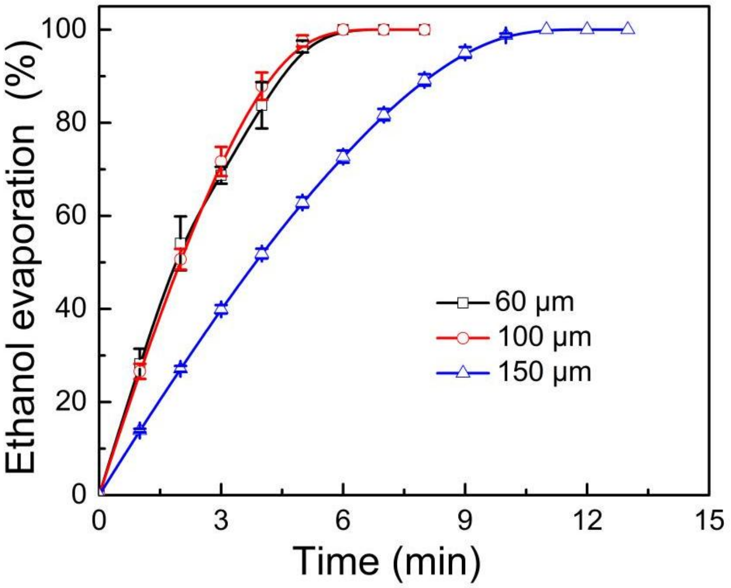

Supplementary Fig. 11. Mass percent of the ethanol evaporation for the porous PP film infiltrated with ethanol as a function of time in open air. Error bars denote the standard deviation.

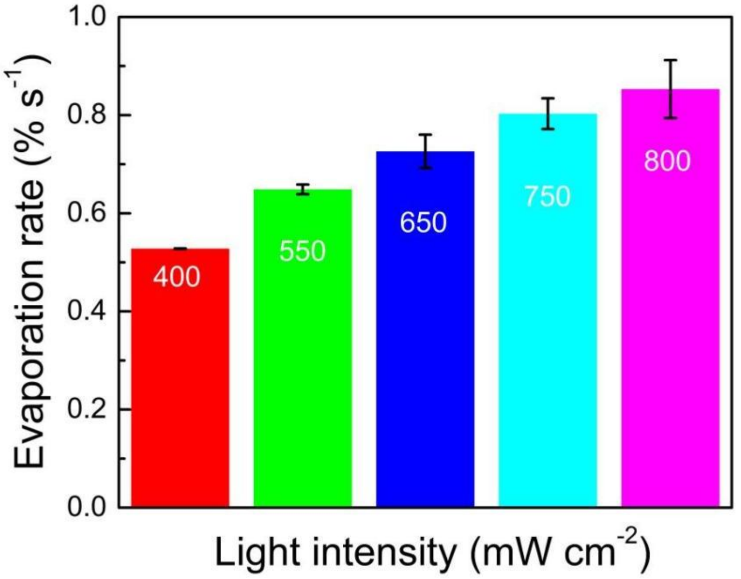

Supplementary Fig. 12. Ethanol evaporation rate for the 100 μm thickness porous PP/CB film under different light intensities. The evaporation rate (ν) was calculated by the percent mass decrease for the porous PP film infiltrated with ethanol divided by the time during complete ethanol evaporation. $v = m_{PP} / ((m_{PP} + m_{ethanol}) \cdot t)$, where m_{PP}, $m_{ethanol}$, and t are the mass of dry porous PP film, the mass of the ethanol for full infiltration, and the time for complete ethanol evaporation. Error bars denote the standard deviation.

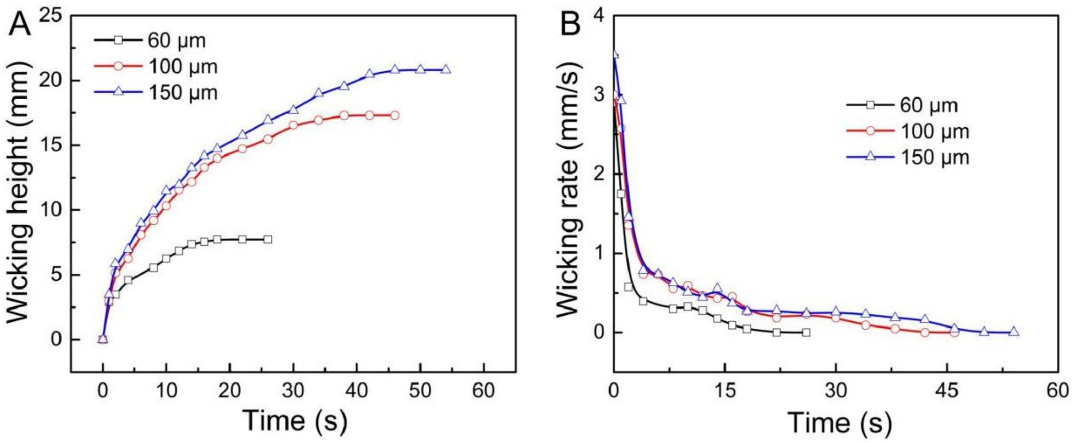

.

Supplementary Fig. 13. Wicking height (A) and wicking rate (B) as a function of time for the porous PP/CB film with different thickness. The original length and width of the film are 5 cm × 1 cm.

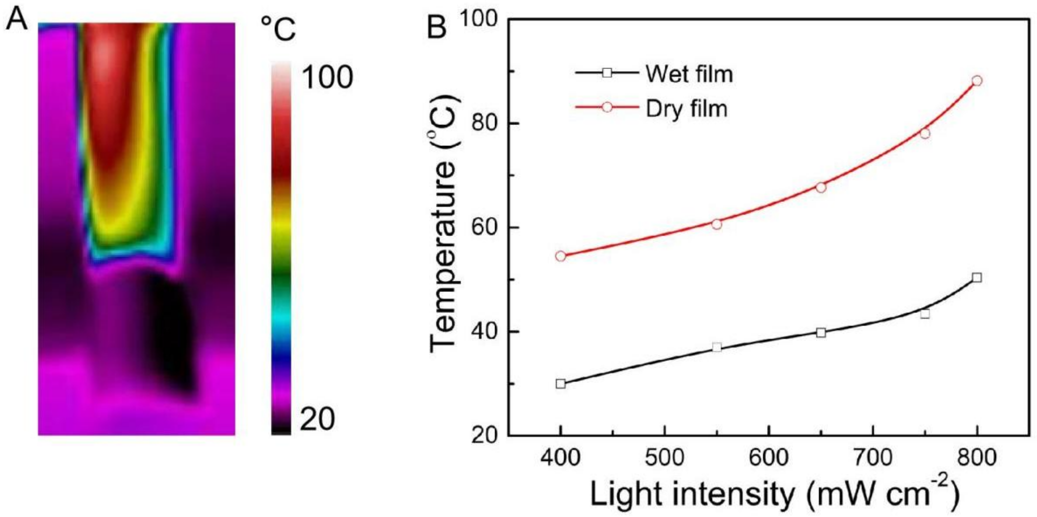

Supplementary Fig. 14. (A) Infrared image of the PP/CB film when infiltrated with ethanol and reached the maximum wicking height at under 800 mW cm^{-2} NIR light. (B) Surface temperature of the wet PP/CB film with ethanol infiltration and dry film without ethanol infiltration at different light intensities.

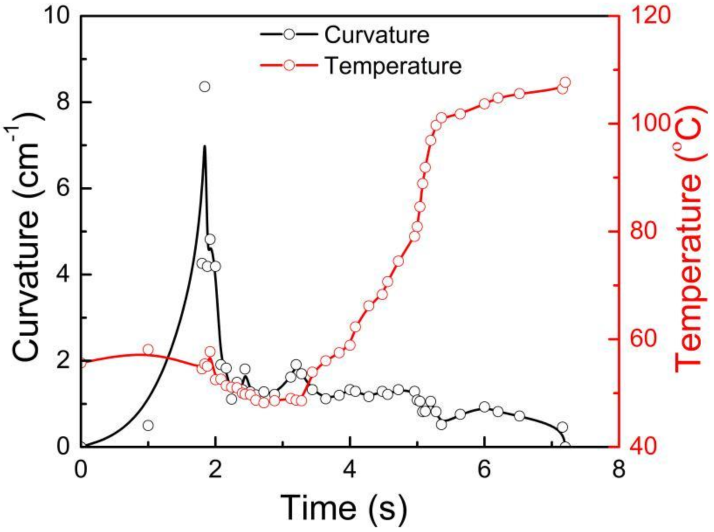

Supplementary Fig. 15. Curvature and temperature as a function of time when the 100 μm thickness PP/CB film infiltrated with ethanol was irradiated by 800 mW cm^{-2} NIR light at a tilt angle of 90°. The original length and width of the film are 9 mm × 3 mm.

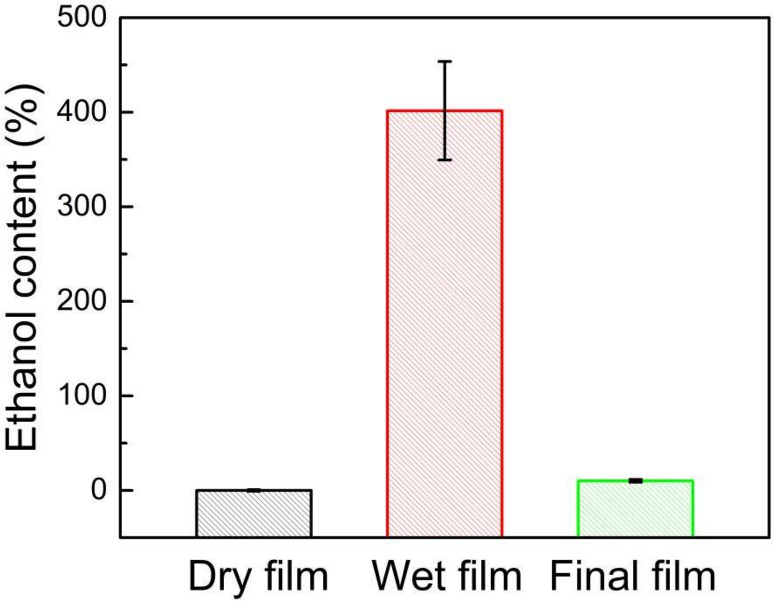

Supplementary Fig. 16. Ethanol content of the100 μm thickness PP/CB film at different states (Dry film indicated the film without ethanol infiltration; Wet film indicated the film with saturated ethanol infiltration; Final film indicated the film stopped actuating under NIR light). The original length and width of the film are 9 mm × 3 mm. Error bars denote the standard deviation.

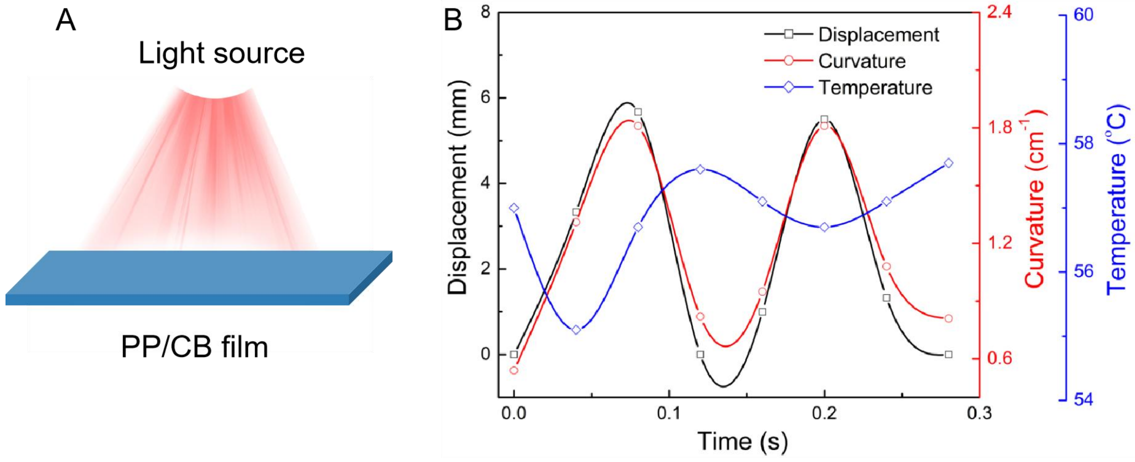

Supplementary Fig. 17. Schematic illustration of the horizontally placed film irradiated vertically by light (A) and Displacement, curvature, and temperature as a function of time for the horizontally placed PP/CB film under 800 mW cm^{-2} NIR light at a tilt angle of 90° (B).

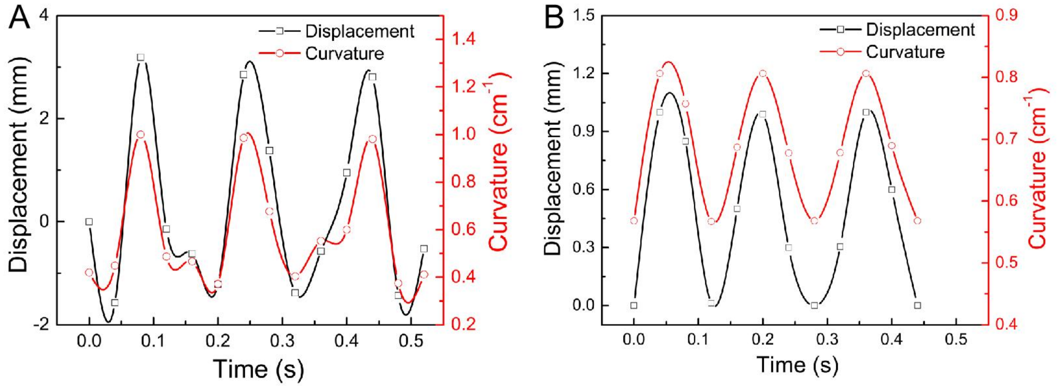

Supplementary Fig. 18. Displacement, and curvature as a function of time for the horizontally placed PP/CB film under 800 mW cm^{-2} NIR light at a tilt angle of 45° (A) and 0° (B).

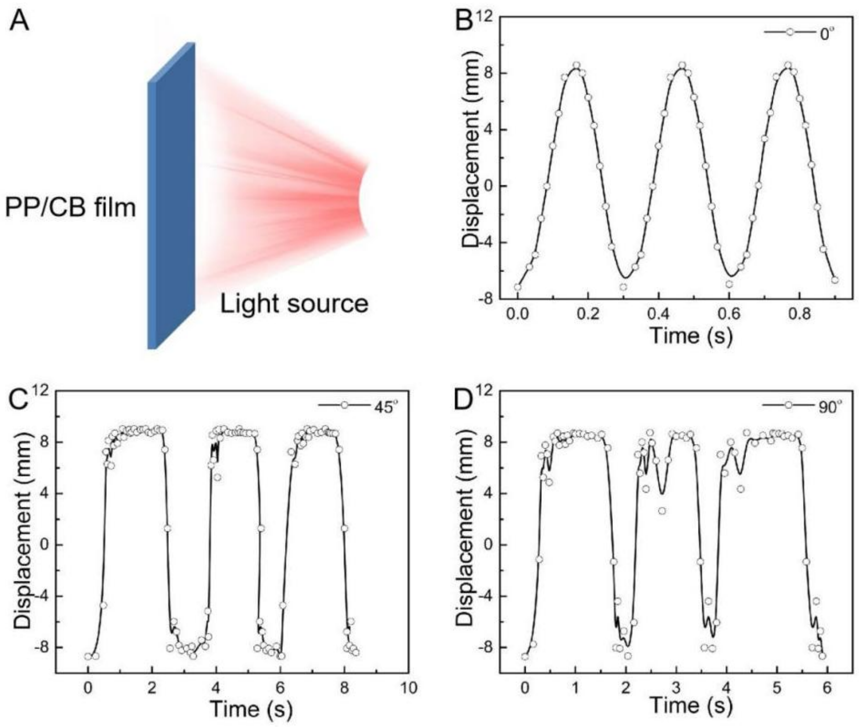

Supplementary Fig. 19. Schematic illustration of the vertically placed film irradiated vertically by light (A) and Displacement as a function of time for the actuator film with 90° tilt angle film under 800 mW cm^{-2} NIR light at a tilt angle of 0° (B), 45° (C), and 90° (D).

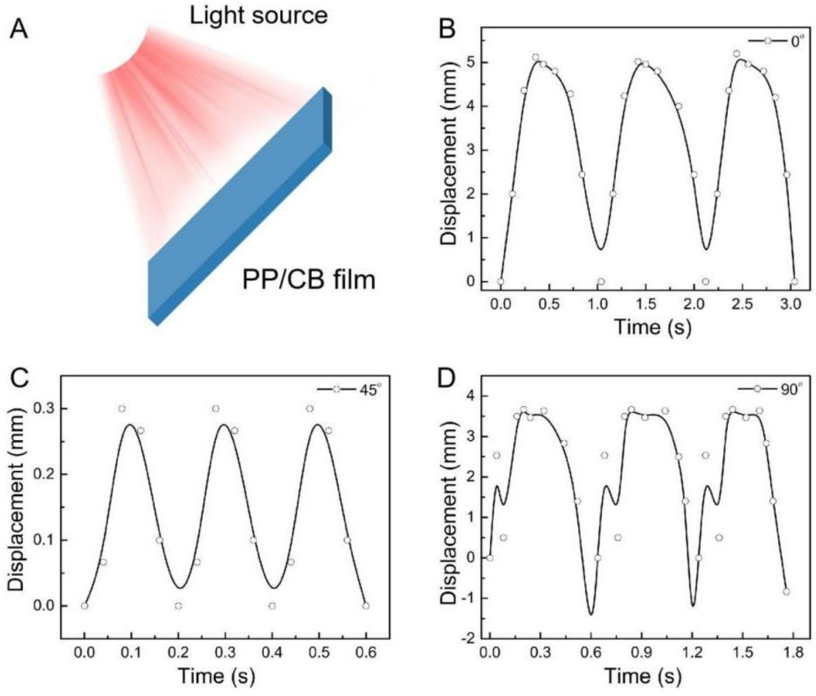

Supplementary Fig. 20. Schematic illustration of the film with 45° tilt angle irradiated vertically by light (A) and Displacement as a function of time for the actuator film with 45° tilt angle film under 800 mW cm^{-2} NIR light at a tilt angle of 0° (B), 45° (C), and 90° (D).

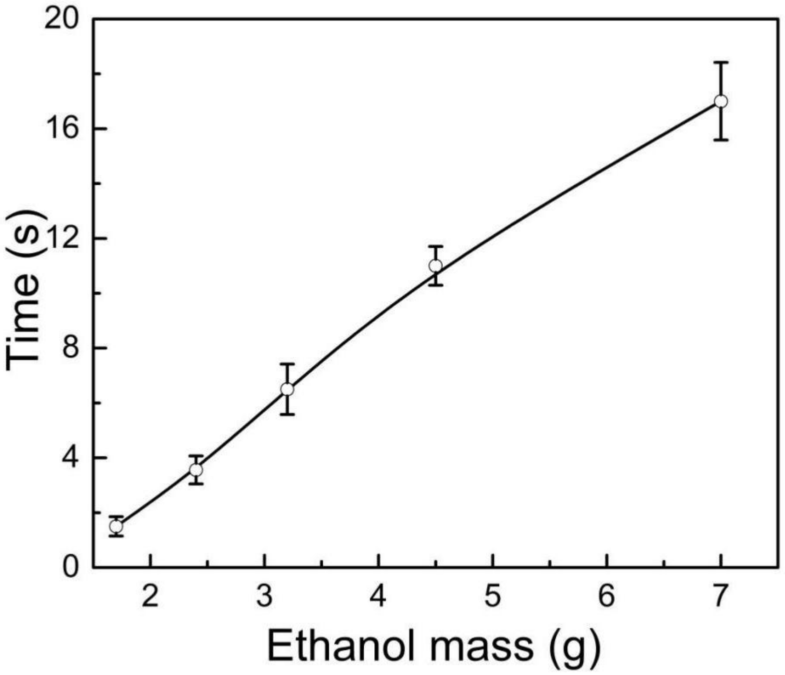

Supplementary Fig. 21. Time as a function of ethanol mass for the PP/CB film reaching to the stable oscillation. The vertically placed porous PP film with original size of 9 mm × 3 mm × 100 μm supplied with ethanol were used as the actuator. The vertically irradiated 800 mW cm^{-2} NIR light were used for actuation by photothermal induced solvent irradiation. Error bars denote the standard deviation.

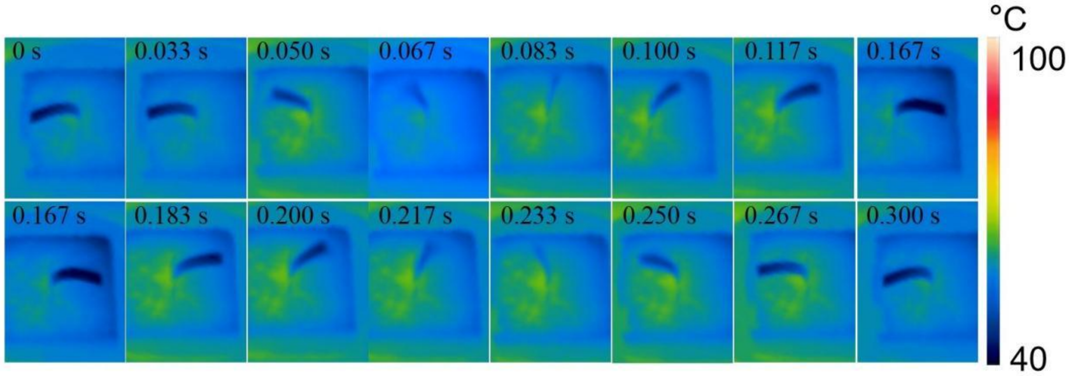

Supplementary Fig. 22. Infrared images at different time for the oscillating bending actuation. The vertically placed porous PP film with original size of 9 mm × 3 mm × 100 μm supplied with ethanol were used as the actuator. The vertically irradiated 800 mW cm^{-2} NIR light were used for actuation by photothermal induced solvent irradiation. If not specified, the same size and configuration of the actuator, and the same light intensity and irradiation angle were used in the following oscillating actuation experiments.

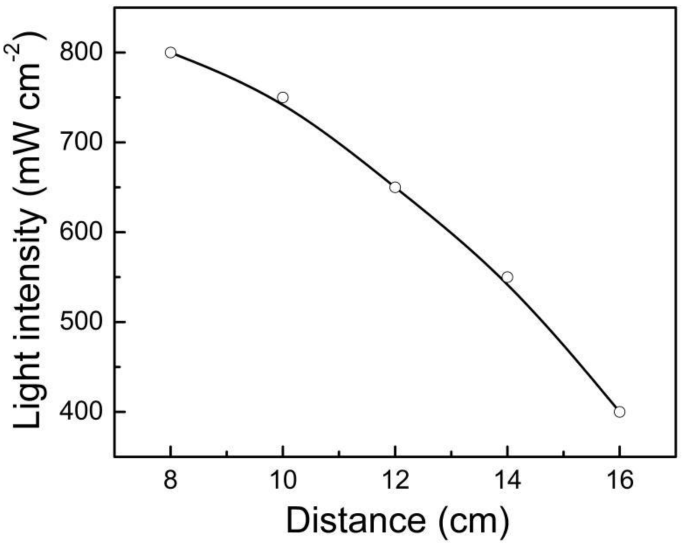

Supplementary Fig. 23. Light intensity as a function of distance of the actuator under NIR light.

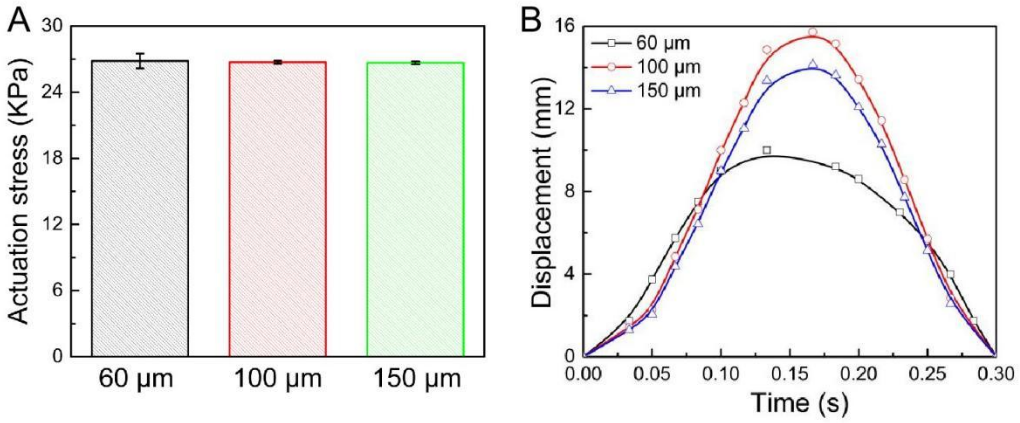

Supplementary Fig. 24. Actuation stress for the porous PP/CB film (2 cm × 1 cm) with different thickness (A), and displacement as a function of time for the oscillating bending actuation under NIR light with 800 mW cm^{-2} for the porous PP/CB film with different thickness (B). The original length and width of the film are 9 and 3 mm, respectively. Error bars denote the standard deviation.

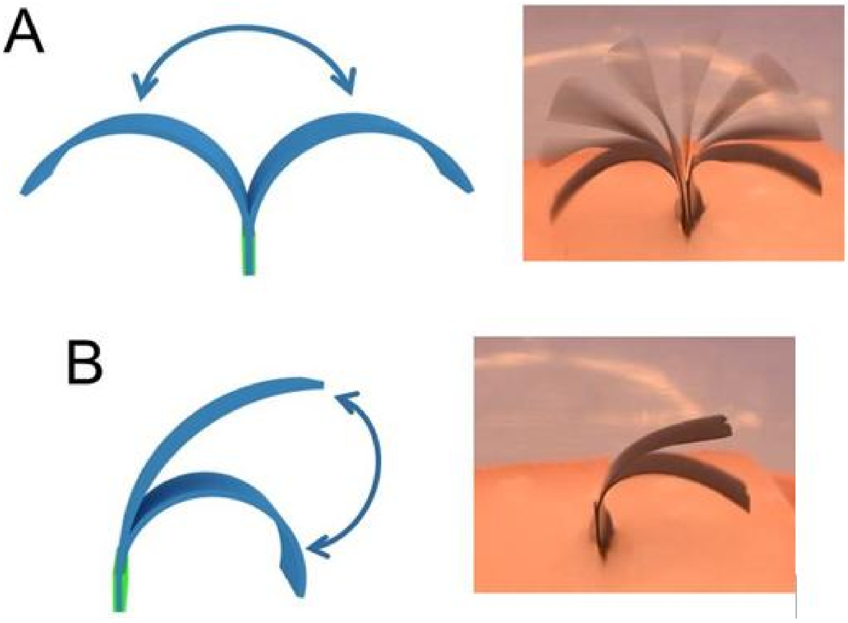

Supplementary Fig. 25. Schematic illustration (left) and photographs (right) for the oscillating actuation under NIR light with 800 mW cm^{-2} (A) and 400 mW cm^{-2} light intensities.

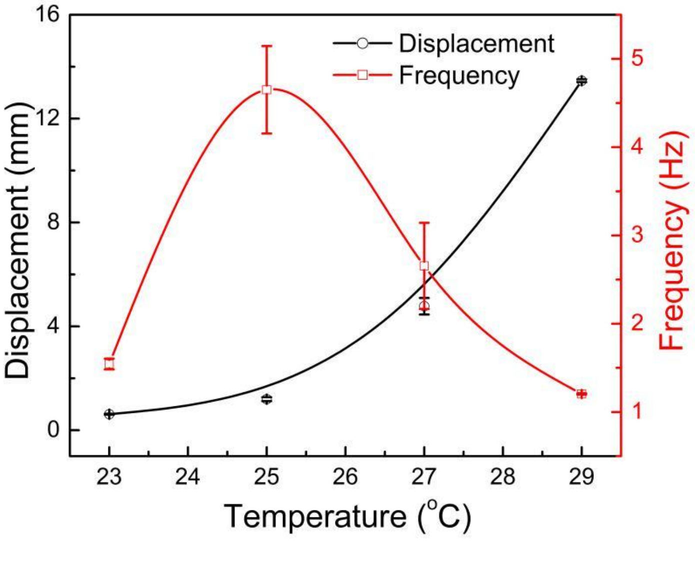

Supplementary Fig. 26. Displacement and actuation frequency of the PP/CB porous film as a function of environmental temperature under NIR light with 400 mW cm^{-2}. The size of the PP/CB film is 9 mm × 3 mm × 100 µm. Error bars denote the standard deviation.

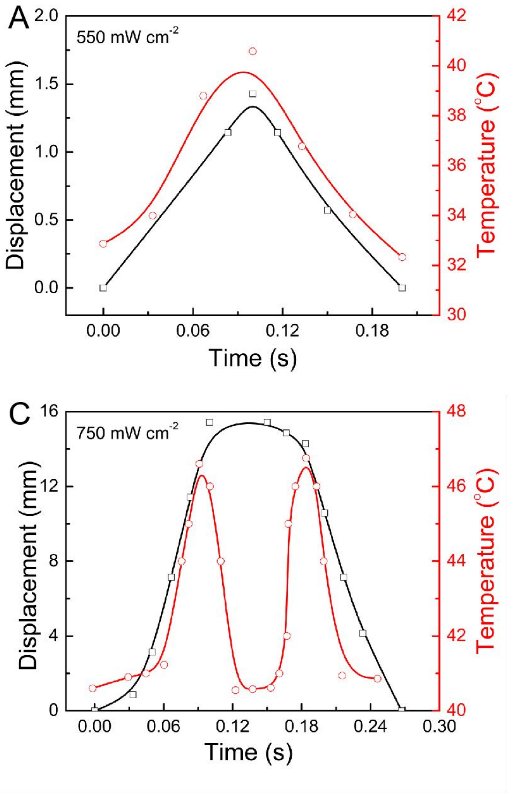

Supplementary Fig. 27. Displacement and maximum surface temperature as a function of time for the oscillating bending actuation under NIR light with 550 mW cm^{-2} (A), 650 mW cm^{-2} (B), and 750 mW cm^{-2} (C).

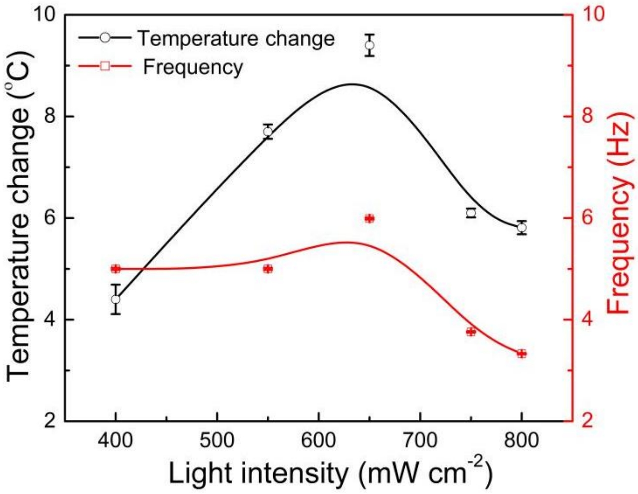

Supplementary Fig. 28. Temperature change and oscillating frequency of the actuator as a function of light intensity. Error bars denote the standard deviation.

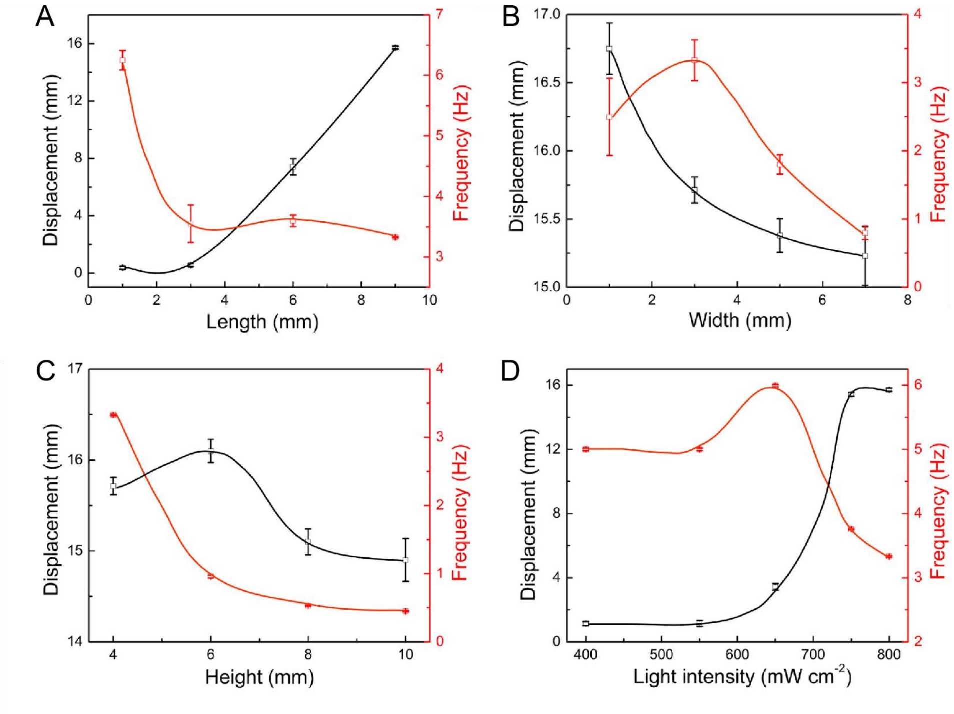

Supplementary Fig. 29. Displacement and oscillation frequency as a function of actuating film with different lengths (A), widths (B), wetting heights of the solvent in the film (C), light intensities (D). The original size of the PP/CB film is 9 mm × 3 mm × 100 μm. Error bars denote the standard deviation.

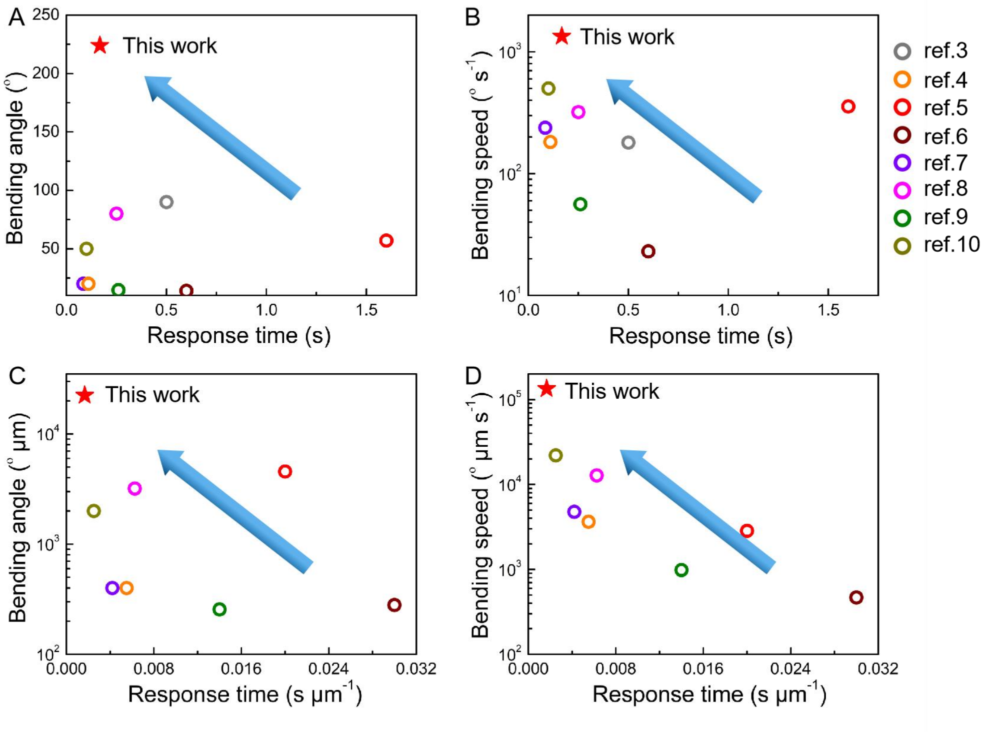

Supplementary Fig. 30. Comparison of the bending angle and response time, bending speed and their values normalized with thickness with other oscillating actuators.

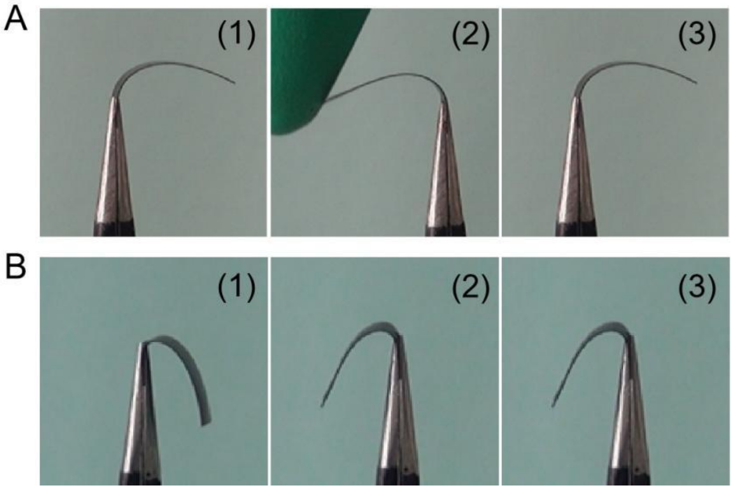

Supplementary Fig. 31. Photographs of the dry PP/CB film (A) and wet PP/CB film infiltrated with ethanol (B) at different states. (1) the initial shape; (2) the bending state under the external force of the finger; (3) the final state on removing the external force.

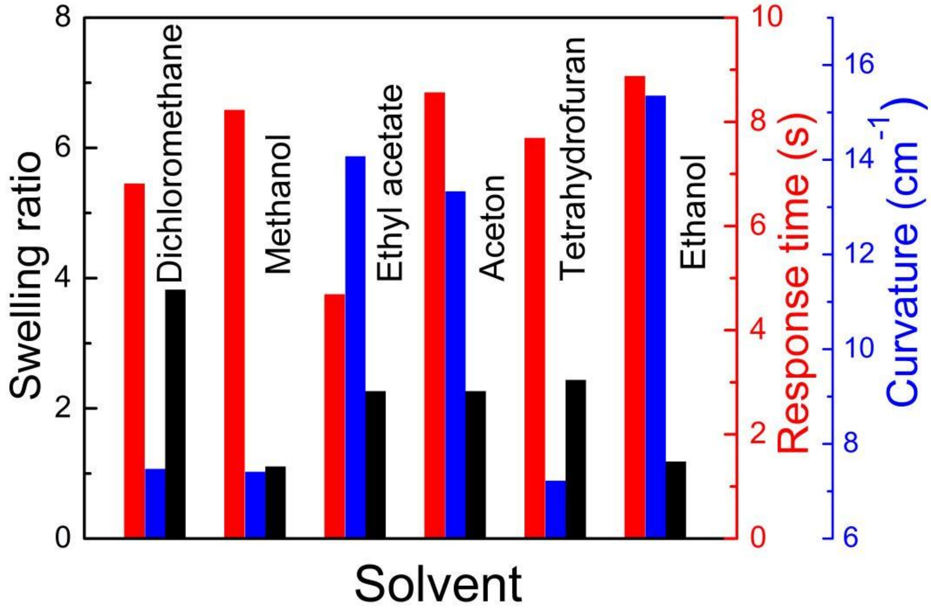

Supplementary Fig. 32. Swelling ratio, response time and curvature of the 100 μm thick film in different solvent systems.

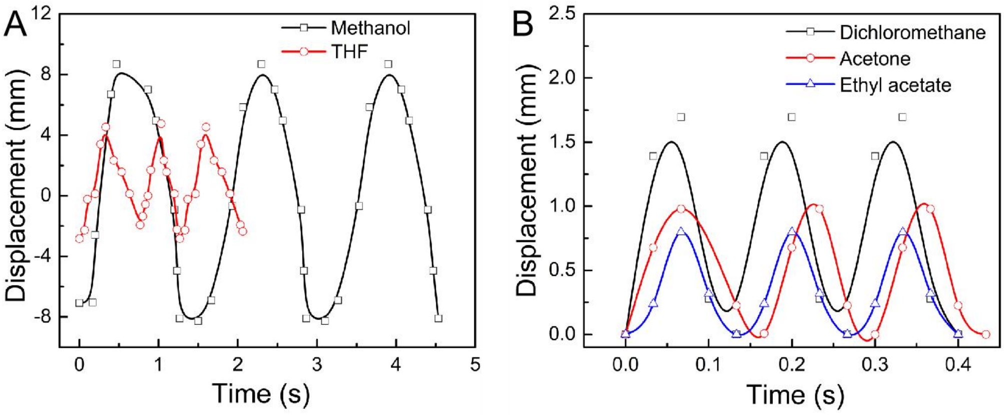

Supplementary Fig. 33. Displacement as a function of time for the oscillating bending actuation under NIR light with different solvents supply, (A) methanol and tetrahydrofuran (THF), (B) dichloromethane, acetone, and ethyl acetate.

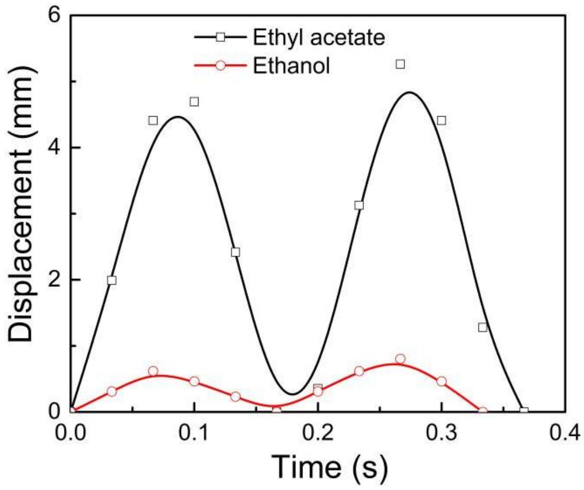

Supplementary Fig. 34. Displacement as a function of time for the porous PDMS film under NIR light with ethanol and ethyl acetate supply. The original dimension of the film is 5 mm × 2 mm × 35 μm.

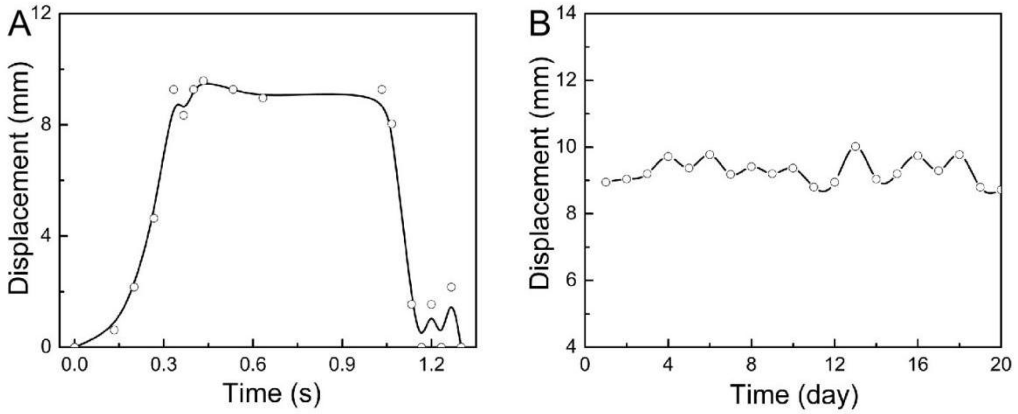

Supplementary Fig. 35. Displacement as a function of time for the porous PP/CB film under the intense light with an irradiation temperature of about 36 °C (A) and the performance stability test for 20 days (B).

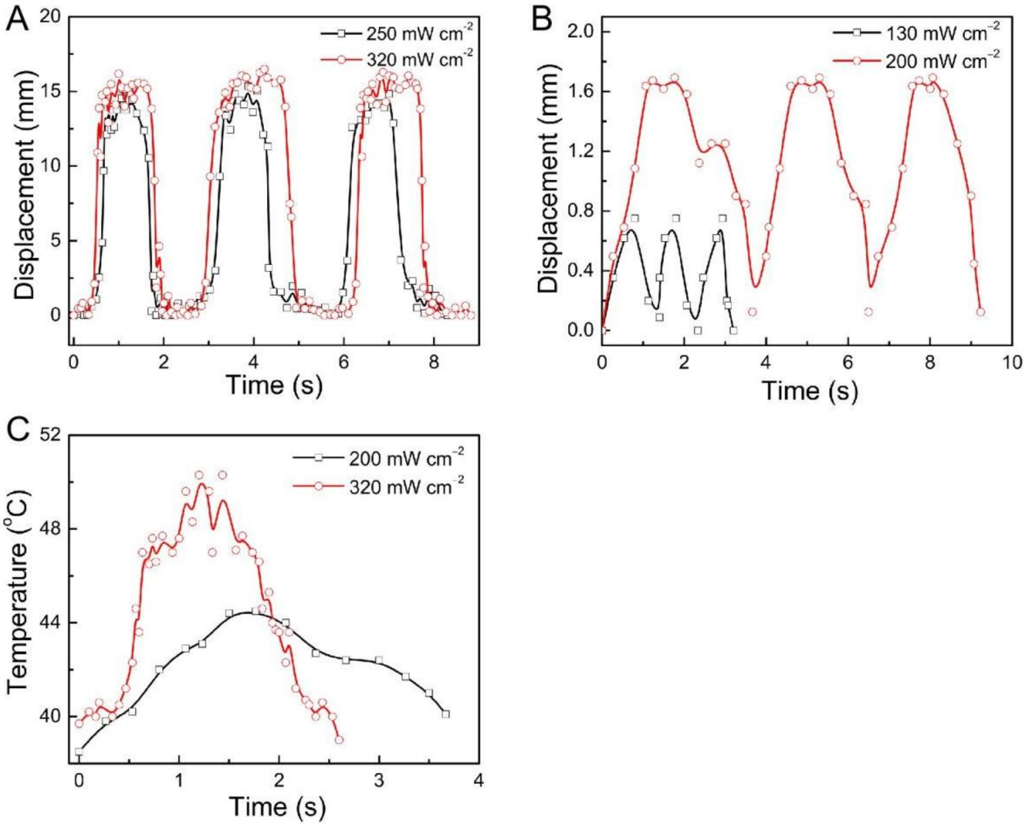

Supplementary Fig. 36. Displacement as a function of time for the oscillating bending actuation under Xe lamp at different light intensities, (A) 250 mW cm^{-2} and 320 mW cm^{-2}, (B) 130 mW cm^{-2} and 200 mW cm^{-2}. (C) Temperature as a function of time for the oscillating bending actuation under Xe lamp at 200 mW cm^{-2} and 320 mW cm^{-2}.

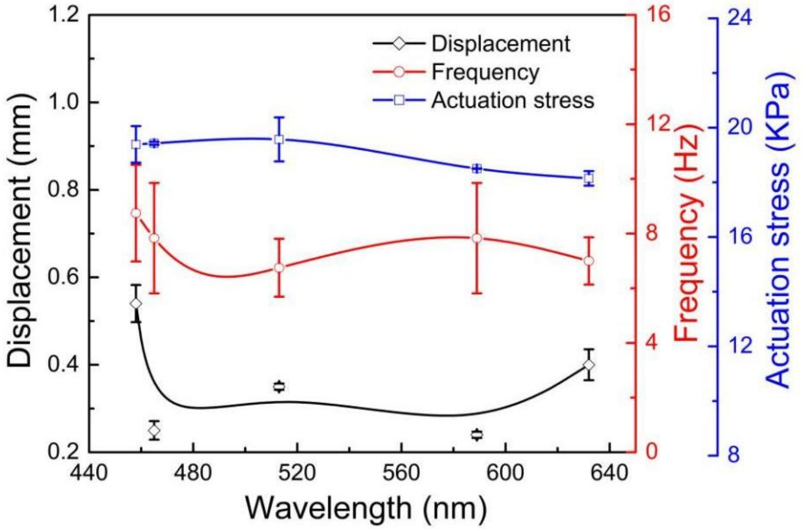

Supplementary Fig. 37. Displacement, actuation frequency, and actuation stress as a function of incident light wavelength. Error bars denote the standard deviation.

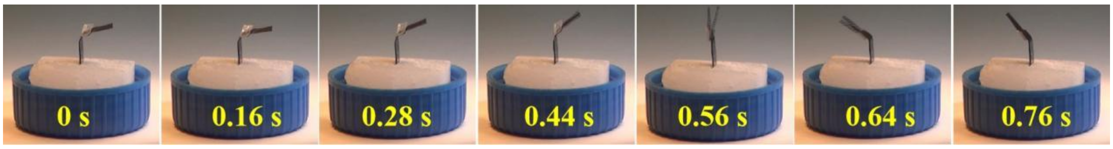

Supplementary Fig. 38. Photographs of the oscillating actuation at different time by carrying 1.9-times load. The original size of the PP/CB is 9.5 mm length and 5 mm width.

Supplementary Table 1. Comparison of the light-induced oscillating bending actuation of this work with those of typical photo-induced oscillators.

Materials	PP/CB (this work)	AuNPs-PNIPAM[3]	F-azo[6]	LC Photonic[8]	CNT/PDMS[5]	PDA-LCN[4]	Azo-LCN-Kapton[9]	M1-LCN[10]
Light source	NIR	Laser	532 nm	Visible	White	NIR	365 nm	UV
Light intensity (mW cm^{-2}/mW)	800/-	-/500	35/-	100/-	330/-	2500/-	100/-	200/-
Thickness d (μm)	100	-	20	40	80	20	17.5	40
Curvature κ/angle θ (cm^{-1}/°)	7.3/224°	1.1/90°	0.2/14°	0.7/80°	0.8/57°	0.3/20°	0.25/14.6°	0.35/50°
$\kappa \times d$ (10^{-4})	730	-	4	28	64	6	4.38	14
Response time t (s)	0.167	0.5	0.6	0.25	1.6	0.11	0.26	0.1
$\theta \times d$ (° μm)	22400	-	280	3200	4560	400	255.5	2000
t/d (s μm^{-1})	0.00167	-	0.03	0.00625	0.02	0.0055	0.0149	0.0025
κ/t (cm^{-1} s^{-1})	43.7	2.2	0.33	2.8	0.5	2.73	0.96	3.5
θ/t (° s^{-1})	1341	180	23.3	320	356.25	182	56.15	500
$\kappa \times d/t$ (10^{-4} s^{-1})	4371	-	6.7	112	40	54.5	16.8	140
$\theta \times d/t$ (° μm^{-1} s^{-1})	134131	-	466.7	12800	2850	3636	982.7	22000
Displacement (mm)	15.7	0.5	3.08	-	13.9	6.7	-	-
D/L	1.74	0.029	0.193	-	1.1	0.67	-	-
T_{max} (°C)	48.5	34.3	31	84.6	99	60	37	-
ΔT (°C)	5.8	3.7	7	58.7	29	35	2.9	-
Frequency (Hz)	6	0.9	0.7	0.15	0.3	9	2.2	5
Divergent light	Yes	Yes	No	No	No	No	Yes	No
Local light	No	No	Yes	Yes	Yes	Yes	Yes	Yes

Au NP-PNIPAM: gold nanoparticles-poly(*N*-isopropylacrylamide); F-azo: ortho-Fluor azobenzene; LCN: liquid crystal polymer network; CNT: carbon nanotube; PDMS: polydimethylsiloxane; PDA: polydopamine.

Supplementary References

1. Han B. et al. Reprogrammable soft robot actuation by synergistic magnetic and light fields. *Adv. Funct. Mater*. **32**, 2110997 (2021).
2. Gelebart, A. H., Vantomme, G., Meijer, E. W. & Broer, D. J. Mastering the photothermal effect in liquid crystal networks: a general approach for self-sustained mechanical oscillators. *Adv. Mater.* **29**, 1606712 (2017).
3. Zhao, Y. S. et al. Soft phototactic swimmer based on self-sustained hydrogel oscillator. *Sci. Robot*. **4**, eaax7112 (2019).
4. Lan, R. C. et al. Near-infrared photodriven self-sustained oscillation of liquid-crystalline network film with predesignated polydopamine coating. *Adv. Mater*. **32**, 1906319 (2020).
5. Yang, L. L. et al. An autonomous soft actuator with light-driven self-sustained wavelike oscillation for phototactic self-locomotion and power generation. *Adv. Funct. Mater*. **30**, 1908842 (2020).
6. Kumar, K. et al. A chaotic self-oscillating sunlight-driven polymer actuator. *Nat. Commun.* **7**, 11975 (2016).
7. Gelebart, A. H., Vantomme, G., Meijer, E. W. & Broer, D. J. Mastering the photothermal effect in liquid crystal networks: a general approach for self-sustained mechanical oscillators. *Adv. Mater.* **29**, 1606712 (2017).
8. Wei, W. Y., Zhang, Z. W., Wei, J., Li, X. F. & Guo, J. B. Phototriggered selective actuation and self-oscillating in dual-phase liquid crystal photonic actuators. *Adv. Optical Mater.* **6**, 1800131 (2018).
9. Wang, J. C., Song, T. F., Zhang, Y. H., Liu, J. G., Yu, M. M. & Yu, H. F. Light-driven autonomous self-oscillation of a liquid-crystalline polymer bimorph actuator. *J. Mater. Chem. C* **9**, 12573 (2021).
10. Sun, J., Hu, W., Zhang, L. Y., Lan, R. C., Yang, H. & Yang, D. K. Light-driven self-oscillating behavior of liquid-crystalline networks triggered by dynamic isomerization of molecular motors. *Adv. Funct. Mater.* **31**, 2103311 (2021).
