# Supplementary material for: Oscillating light engine realized by photothermal solvent evaporation
Source: Nat Commun. 2022 Sep 24;13:5621. doi: 10.1038/s41467-022-33374-x (PMC9509359; doi:10.1038/s41467-022-33374-x)
Supplement: Supplementary file 3 — Description of Additional Supplementary Files [file 41467_2022_33374_MOESM3_ESM.pdf]

Description of Additional Supplementary Files

File Name: Supplementary Movie 1.

Description: Oscillating bending actuation of the vertically placed porous PP film. The original size of the actuator was 9 mm × 3 mm × 100 μm. The actuator was supplied with ethanol and under vertically irradiated 800 mW cm^{-2} NIR light. The maximum bending curvature and oscillating displacement are 7.3 cm^{-1} and 15.7 mm, respectively.

File Name: Supplementary Movie 2.

Description: Oscillating bending actuation of the vertically placed porous PP film carrying a load 6-times its own mass. The original size of the actuator was 9.5 mm × 5 mm × 100 μm. The actuator was supplied with ethanol and under vertically irradiated 800 mW cm^{-2} NIR light. The maximum specific work and specific power are 12.02×10^{-5} J g^{-1} and 2×10^{-4} W g^{-1}, respectively.
